# Impact of transsexualizing process centers on self-medication of transgender individuals

**DOI:** 10.11606/s1518-8787.2024058005791

**Published:** 2024-07-10

**Authors:** Arthur Machado Geiger Dias de Moraes, Caren Nariel Pereira Santos Souza, Luiza Taddeo Marques, João Fernando Nascimento de Barcelos, Felipe Barros Oliveira, Rafaela Góes Bispo, Rodrigo Gomes Santos, Ailton da Silva Santos, José Antônio Diniz Faria, Luciana Mattos Barros Oliveira

**Affiliations:** I Universidade Federal da Bahia Instituto de Ciências da Saúde Departamento de Biorregulação Salvador BA Brasil Universidade Federal da Bahia. Instituto de Ciências da Saúde. Departamento de Biorregulação. Salvador, BA, Brasil; II Centro Estadual Especializado em Diagnóstico, Assistência e Pesquisa Salvador BA Brasil Centro Estadual Especializado em Diagnóstico, Assistência e Pesquisa. Salvador, BA, Brasil

**Keywords:** Health Services for Transgender Persons, Transgender Persons, Self Medication, Hormone Therapy

## Abstract

**OBJECTIVE:**

The transgender population in Brazil faces marginalization and difficulties in accessing education and health, leading many individuals to self-medicate. This study aimed to evaluate the impact of the implementation of Specialized Centers in the Transsexualizing Process (SCTP) on the use of cross-sex hormone therapy (CSHT) without medical prescription, as well as the level of education and mental health profile of these individuals.

**METHODS:**

This is a cross-sectional study with data from physical and electronic medical records between September 2017 and February 2023 regarding the use of CSHT before and after the implementation of two SCTP in the state of Bahia, Brazil, in addition to data on education level, previous diagnosis of anxiety and depression of patients.

**RESULTS:**

A total of 219 participants, 127 transgender men (TM) and 92 transgender women and *travestis* (TrTW), were assessed. A significant reduction in the prevalence of self-medication was observed in both TrTW (92.98% before and 51.43% after, p<0.001), and TM (47.17% before and 25.67% after, p = 0.010) with the implementation of SCTP. Transgender individuals who used CSHT before accessing the service were found to have a lower prevalence of depression. Self-medication was not significantly associated with education or anxiety in our sample.

**CONCLUSION:**

The results indicate the need for the expansion of SCTP, as they were associated with lower rates of self-medication in the transgender population.

## INTRODUCTION

The transgender population, which includes individuals who do not identify with the biological sex assigned at birth, is commonly exposed to the consequences of prejudice and marginalization imposed by a heteronormative society^1^. Trans men (TM) are those individuals who have a female sex assigned at birth but a male gender identity. Trans women and *travestis* (TrTW), on the other hand, are those whose biological sex is male, but who have a female gender identity. The differences between transgender women and *travestis* are mainly political and self-identified. This affects access to health services, which encompass all the multiprofessional care required for this population^2^. Thus, when patients reach a specialized service, they frequently arrive already using sex hormones on their own, potentially damaging their health.

Facilitating access to multiprofessional care, especially when available through the Brazilian Unified Health System (SUS), allows individualized and continuous support for cross-sex hormone therapy (CSHT), psychosocial support and other health services involved in the process^2^.

Ordinance No. 2,803/2013 of the Ministry of Health, which “redefined and expanded the Transsexualizing Process in the Unified Health System (SUS)”, was the first to cover the Transsexualizing Process more democratically, enabling the implementation of Specialized Centers (SCTP) in several states of Brazil^2^. In addition, the Federal Council of Medicine Resolution No. 2,265/2019 provides for the expansion of access to care for this population, establishing criteria for greater safety in hormone therapy and gender-affirming surgeries^3^. These regulations were important because they enabled the inclusion of all procedures and respective follow-up protocols, whether psychological, hormonal, or even surgical, in the Unified Health System, in addition to standardizing treatment for children and adolescents and adopting the singular therapeutic process as a precursor to transgender care.

In Brazil, access to education by transgender people, especially at the higher education level, is impaired by stigma and prejudice^4^. For these individuals, the school environment fosters insecurity regarding sexual orientation and gender expression.^5^ This scenario culminates in restricted access of transgender people to higher education, as shown by the data of Scote et al., in which 59.4% of the *travestis* interviewed had completed high school, while 6.5% had incomplete higher education and the minority (2.2%) had completed higher education^6^. Krüger et al. showed that the search for medical orientation regarding the use of hormones by transgender women is directly related to income and education^7^.

The objective of this study was to evaluate, in Bahia, Brazil, the impact of the implementation of two Specialized Centers in the Transsexualizing Process (SCTP) in 2017 and 2018 on the use of cross-sex hormone therapy (CSHT) without medical prescription, as well as the level of education and mental health profile of these individuals.

## METHODS

### Study Design and Participants

This is a cross-sectional study with data from physical and electronic medical records of transgender patients who sought specialized medical care to start cross-sex hormone therapy in at least one of the two SCTPs in the state of Bahia, Brazil, between September 2017 and February 2023. The inclusion criteria were as follows: 1) self-identified TrTW or TM; 2) search for gender adequacy through hormone therapy; 3) presentation of a mental health evaluation made by a psychiatrist or psychologist; 4) age over 18 years. Non-binary transgender individuals were not included in this analysis.

### Variables and Measurements

Data collected for this study were gender identity, previous hormone use, date of cross-sex hormone therapy onset, level of education, and report of previous diagnosis of anxiety and depression by a mental health professional, as seen in Table 1. The self-medication group consisted of patients who reported using CSHT on their own. As for the group without self-medication, of those who reported never having done CSHT or having done it under medical guidance.

**Table 1:** Each measured variable and its respective value/category.

Variable	Value/Category
Gender identity	Transgender male; transgender female, and *travestis*
Previous hormone use	Self-medication; Health care professional follow-up
Date of CSHT onset	Date (MM-YYYY)
Level of education	Illiterate; Complete primary; Incomplete high school; Complete high school
Previous diagnosis of anxiety or depression	None; Anxiety; Depression; Both

**Figure 1:**
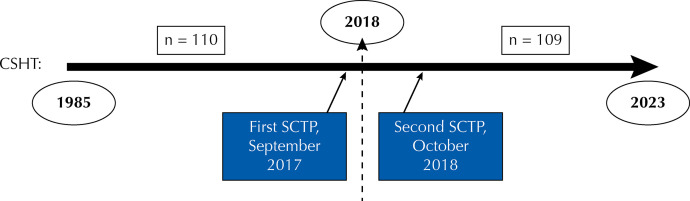
Timeline of the start date of cross-sex hormone therapy and SCTP implementation.

CSHT: cross-sex hormone therapy; SCTP: Specialized Centers in the Transsexualizing Process.

January 1^st^, 2018 was defined as the cut-off point for regular endocrinologic care at SCTP. Patients were divided into two groups: those who started CSHT before 2018 (n = 110) and those who started from 2018 (n = 109). Figure 1 shows a timeline from 1985 (earliest start date of CSHT) to 2023, according to the start date of hormone therapy, as well as the time of SCTP implantation in September 2017 and October 2018.

### Ethical Aspects

The research methods were reviewed and approved by the Research Ethics Committee (REC) of both institutions, and all participants provided verbal and written authorization in the Informed Consent Form (ICF), which guaranteed anonymity and confidentiality of the information provided to the study.

### Statistical Methods

Data were tabulated in Microsoft Excel^®^ 2021 (ver. 2209) and analyzed in RStudio^©^ 2022.12.0, build 353. Statistical analysis was performed using Fisher’s exact test to determine significance, considering values of p < 0.05 significant.

## RESULTS

A total of 219 patients (127 TM and 92 TrTW) were evaluated in this study. After dividing the sample, 53 TM and 57 TrTW were in the group “before 2018” and 74 TM and 35 TrTW in the group “from 2018”.

**Figure 2:**
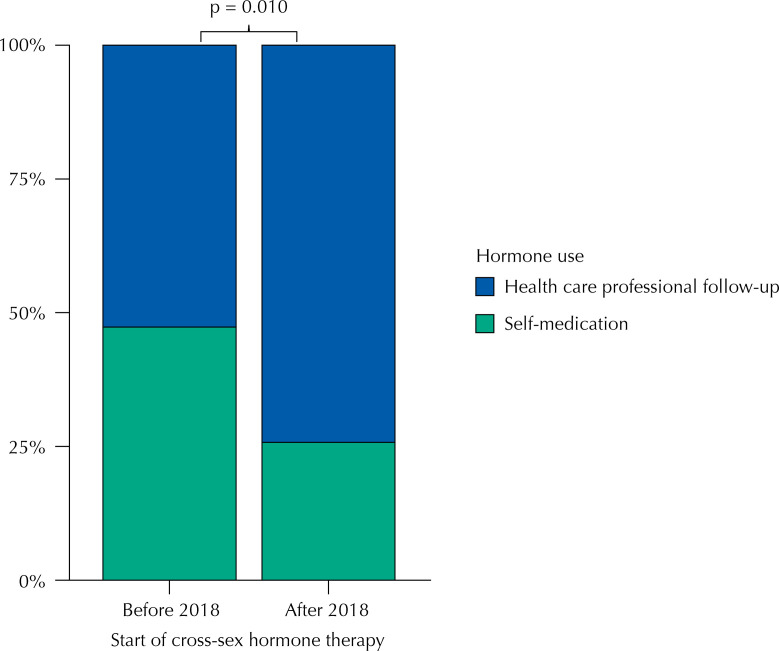
Distribution of transgender men with and without self-medication before and after 2018.

For the TM group, it was found that, before 2018, 47.17% (25 of 53 TM) came to one of the services using cross-sex hormone therapy on their own, while, from 2018, this percentage dropped to 25.67% (19 of 74 TM) (p = 0.010), as shown in Figure 2.

**Figure 3:**
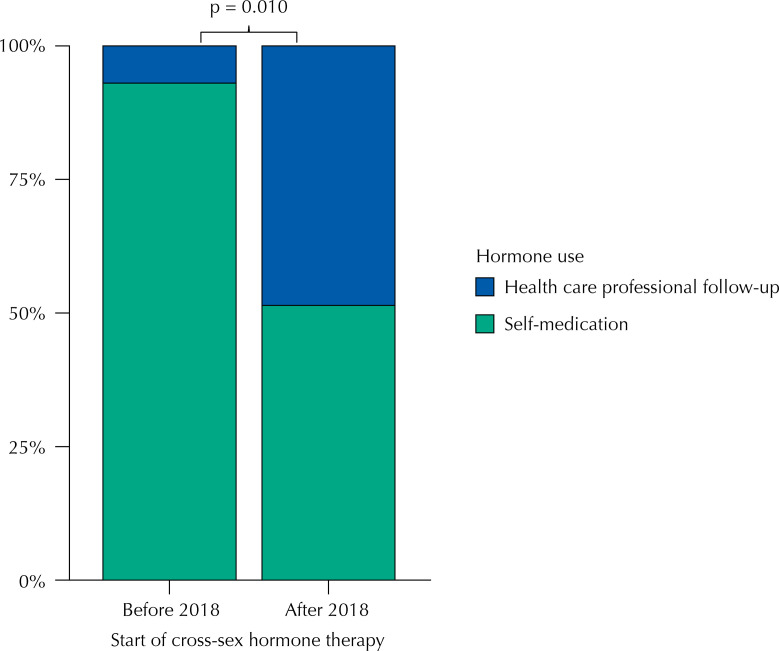
Distribution of transgender women and travestis with and without self-medication before and after 2018.

For the TrTW group, it was found that, before 2018, 92.98% (53 of 57 TrTW) arrived at the service already using cross-sex hormone therapy, whereas after 2018, this percentage dropped to 51.43% (18 of 35 TrTW) (p < 0.001), as shown in Figure 3.

In the analysis of the level of education and prevalence of previous diagnosis of anxiety or depression, it was observed that transgender individuals who used CSHT before accessing the service had a lower prevalence of depression. Twelve percent of those who self-medicated had a previous diagnosis of depression, whereas 27% of those who did not self-medicate had the same diagnosis (p = 0.029). The most prevalent education levels were complete high school (TrTW = 28,7%, TM = 42,9%) and incomplete higher education (TrTW = 25.5%, TM = 26.0%), as shown in Table 2. No correlation was found between self-medication and the level of education or previous anxiety diagnosis.

**Table 2:** Education and previous diagnosis of anxiety and depression in participants with and without self-medication.

Variable	Without self-medication (n = 101^a^)	With self-medication (n = 110^a^)	p-value^b^
Level of education			0.081
Illiterate	1 (1.0%)	2 (1.8%)	
Complete primary education	1 (1.0%)	8 (7.3%)	
Incomplete high school education	14 (14%)	21 (19%)	
Complete high school education	39 (39%)	44 (40%)	
Incomplete higher education	28 (28%)	21 (19%)	
Complete higher education	18 (18%)	12 (11%)	
Unknown	0 (0%)	2 (1.8%)	
Anxiety			0.066
No	43 (65%)	57 (79%)	
Yes	23 (35%)	15 (21%)	
Unknown	35	38	
Depression			0.029
No	48 (73%)	63 (88%)	
Yes	18 (27%)	9 (12%)	
Unknown	35	38	

^a^ n (%).

^b^ Fisher’s exact test.

## DISCUSSION

In the present study, a reduction in self-medication was observed after the implementation of SCTP at Salvador, Bahia, Brazil. Transgender people who used CSHT before accessing the service had a lower prevalence of previous diagnosis of depression, but self-medication did not correlate with level of education or previous diagnosis of anxiety. In this sample, the most prevalent levels of education were complete high school education and incomplete higher education.

Self-medication is an extremely common problem among Brazilians, with a prevalence of 16.1%, and is more frequent in females and in patients with chronic diseases^8^. Although there are no studies in the literature evaluating the prevalence of self-medication in the transgender population of Brazil as a whole, the data from the present study indicate that, regarding only cross-sex hormone therapy, self-medication in transgender patients could already be more frequent than that found in the general population^8^.

In a study conducted by Mepham et. al. at the National Nottingham Gender Clinic, of the 145 transgender participants, 23% stated that they had already self-medicated at the time of the interview, which was approximately 5 times more frequent among transgender women than among transgender men^9^. Likewise, in the present findings, higher numbers of self-medication were observed among transgender women (before 2018, 47.2% of TM and 92.98% of TrTW self-medicated; after 2018, self-medication decreased to 25.67% of TM and 51.43% of TrTW)^9,10^. A possible reason for this distinction is the difference in access to hormone therapy, testosterone being harder to obtain because it is an anabolic drug and therefore dispensed in Brazil only with medical prescription, although some transgender men still buy it in the clandestine market^11^. According to Krüger et al, 84% of transgender women and *travestis* use sex hormones without a medical prescription, and instructions regarding the use of these substances are mainly provided by their peers^7^. Mepham et al. showed that 69% of transgender patients who self-medicated had the internet as their primary source of hormones^9^. Since the internet is an important source of information on how to self-medicate, it is essential to consider greater dissemination of SCTP in the virtual environment to improve access to this population and combat this practice.

Although Scote et al.^6^ reported that 59.4% of the *travestis* (TrTW) interviewed had completed high school education and only 6.5% had an incomplete higher education, in the present study, the most prevalent levels of education among TrTW were complete high school education (28.7%) and incomplete higher education (25.5%). Among the TM, 42.9% had completed high school education and 26.0% had an incomplete higher education at the time of the interview. A possible explanation for the higher level of education in the transgender population in the current study is the fact that individuals with higher education seek more medical services such as SCTP and medical guidance on the use of hormones^5,7^. Furthermore, this result may reflect a selection bias, given that the services for the transgender population in Bahia, Brazil, have been recently implemented and their existence is not yet widely known. The search for them is possibly concentrated in transgender people who have a higher level of education and, therefore, more access to information.

Some of the obstacles to transgender women seeking health services in this study may be related to one of the SCTP being in a sexually transmitted infection treatment center. Given that stigma and discrimination have a strong impact on the lives of patients with the disease, the fear of being framed as “HIV positive” could have impaired the access of transgender women to the health service^12^. Another issue is the requirement of prior psychological evaluation to initiate hormone therapy in the context of poor access to health services^13^. In addition, excess estradiol is positively related to erectile dysfunction and may impact the sexual health of TrTW that use the penis in sexual relations^11,14^.

The reasons for the disengagement of not only transgender women, but also transgender men from health services are complex and diverse, including insufficient referral services, poor training of primary care professionals, and a lack of respectful and trans-aware doctors^14^. The difficulties encountered by these individuals when they need access to health services range from disrespect for their social name by receptionists and health professionals to possible discriminatory attitudes based on moral and religious reasons^17^.

According to Ordinance 2803/2013, the provision of hormone therapy by the Brazilian Unified Health System (SUS) takes place exclusively through accredited Specialized Centers in the Transsexualizing Process (SCTP). Therefore, the existence of these centers and their geographical distribution directly impacts the possibility of free and safe hormone therapy for this population^2^.

Another reason for self-medication among these patients is the desire for a faster transition, which possibly accounts for the use of hormones in higher doses than recommended in medical practice^13^. The potential adverse effects of cross-sex hormone therapy are numerous, especially when there is no medical follow-up. While in transgender men, testosterone use can cause arterial dysfunction, dyslipidemia, and hypertension, in transgender women, hormone therapy is related to thromboembolism, myocardial infarction, and ischemic stroke, effects that are directly associated with the dose and route of administration of the drugs^18,19^.

In addition, considering that adherence to cross-sex hormone therapy, an important factor for its effectiveness, was associated with contact with a welcoming and trained health professional, the importance of training professionals who can provide quality care becomes noticeable^13^. In this sense, another benefit of SCTP is the possibility of continuing training for professionals who are already working with the transgender population, especially since the high flow of patients enables the integration of knowledge about the transgender population into the training of health professionals.

Although the use of cross-sex hormone therapy without medical guidance implies several health risks, in the present study, we observed that depression was less prevalent among transgender people who used cross-sex hormone therapy before accessing the specialized service. This finding is in line with other studies in the literature showing evidence that cross-sex hormone therapy reduces the risk of depression in the transgender population^20^. In a study by Aldridge,Z. et al., a statistically significant reduction in depressive symptoms after CSHT was demonstrated, but not in relation to anxiety, which is also in agreement with the findings of the present study^21^. A possible explanation for this is the improvement of mental health in transgender people after body modifications that lead to better adaptation of the body to their gender identity^23^.

This study presented limitations regarding its design (cross-sectional) with the risk of recording bias and the sampling method (non-probability sample) with the risk of selection bias due to the geographical proximity of the two centers. Furthermore, this study did not characterize the participants’ hormone therapy: the dose, time of use, and morbidity of the self-medication were not analyzed. In addition, no specific tests were applied to quantify the rates of depression and anxiety. Data regarding previous diagnosis of anxiety or depression were based on the participants’ reports.

## CONCLUSIONS

The present study demonstrated a significant reduction in the use of cross-sex hormone therapy, without medical prescription, after the implementation of two SCTPs in the state of Bahia, Brazil, and that the adaptation of the body to gender identity by cross-sex hormone therapy, even without medical prescription, is related to a lower prevalence of depression in this population. The data obtained suggest the need for an increasing coverage of SCTP because the access of this population to care and assistance in the transsexualizing process within the Brazilian Unified Health System was associated with a decrease in self-medication rates.
